# Dietary Mapping of Macronutrient Consumption Before Pregnancy Related to Gestational Diabetes Mellitus

**DOI:** 10.3390/nu17203256

**Published:** 2025-10-16

**Authors:** Antigoni Tranidou, Aikaterini Apostolopoulou, Antonios Siargkas, Emmanuela Magriplis, Ioannis Tsakiridis, Georgia Koutsouki, Michail Chourdakis, Themistoklis Dagklis

**Affiliations:** 1 3rd Department of Obstetrics and Gynecology, School of Medicine, Faculty of Health Sciences, Aristotle University of Thessaloniki, 541 24 Thessaloniki, Greece; antigoni.tranidou@gmail.com (A.T.); antonis.siargkas@gmail.com (A.S.); igtsakir@auth.gr (I.T.); koutsoukig@gmail.com (G.K.); 2 Laboratory of Hygiene, Social & Preventive Medicine and Medical Statistics, School of Medicine, Faculty of Health Sciences, Aristotle University of Thessaloniki, 541 24 Thessaloniki, Greece; katapost@yahoo.gr (A.A.); mhourd@auth.gr (M.C.); 3 Department of Food Science and Human Nutrition, Agricultural University of Athens, Iera Odos 75, 118 55 Athens, Greece; emagriplis@eatsmart.gr

**Keywords:** maternal diet, pre-pregnancy nutrition, macronutrients, energy intake, food components, dietary mapping, gestational diabetes mellitus, weighted quantile sum regression, BORN 2020 cohort

## Abstract

**Background/Objectives**: Gestational diabetes mellitus (GDM) is a common pregnancy complication, and maternal diet before conception may be an important modifiable risk factor. This study aimed to investigate the association between maternal pre-pregnancy energy and macronutrient intake and the risk of GDM. **Methods**: Data from the BORN2020 cohort in Northern Greece were used. Women were recruited at their first trimester prenatal visit (11–13 weeks of gestation) and provided detailed dietary data via a validated food frequency questionnaire (FFQ), reflecting intake in the six months prior to conception. Weighted Quantile Sum (WQS) regression models were applied to assess the joint effects of food-derived macronutrient mixtures on GDM risk. Analyses were adjusted for parity, maternal age, pre-pregnancy BMI, thyroid disorders, smoking, energy intake, and physical activity. **Results**: In total, 797 women were included in the analyses. In normal-BMI women, higher pre-pregnancy intake of energy (aOR = 81.16, 95% CI: 4.55–1447.46), total carbohydrates (aOR = 50.66, 95% CI: 3.59–715.04), total fat (aOR = 9.35, 95% CI: 1.17–74.54), and total protein (aOR = 11.06, 95% CI: 1.14–107.09) were significantly associated with increased odds of GDM. The main contributing foods were energy-dense and processed items such as puff pastry, processed meats, potatoes, refined grains, and dairy products. In contrast, dietary fiber, vegetable protein, and animal protein were not significantly associated with GDM risk. Among overweight and obese women, none of the macronutrient or energy mixtures showed significant associations. **Conclusions**: For women of normal weight, a pre-conception diet high in energy and macronutrients from processed foods is a significant predictor of GDM risk. This association was not found in overweight or obese women, highlighting a critical window for targeted nutritional intervention for normal-weight individuals before pregnancy.

## 1. Introduction

Gestational diabetes mellitus (GDM) is a significant public health challenge, defined as glucose intolerance first recognized during pregnancy; its prevalence is rising significantly in line with trends in obesity and increasing maternal age, affecting approximately 14% of pregnancies worldwide [1]. The prevalence is also substantial and rising in the developed world, with estimates of around 11.8% in the United States and 10.9% in Europe [2,3].

GDM develops when the mother’s pancreas cannot produce enough insulin to overcome the natural insulin resistance that occurs during pregnancy, a state induced by placental hormones to ensure glucose supply to the fetus [4]. The consequences may be severe for both mother and child; mothers face a higher risk of preeclampsia, cesarean delivery, and a dramatically increased long-term risk of developing type 2 diabetes and cardiovascular disease [5,6]. Offspring are at risk for excessive birthweight (macrosomia), neonatal hypoglycemia, and a long-term predisposition to obesity and type 2 diabetes in their own lives [5,6]. As many risk factors for GDM are non-modifiable, diet remains a cornerstone of prevention and management, with lifestyle changes being sufficient for glycemic control in up to 85% of cases [7].

Appropriate macronutrient intake is crucial for both maternal health and fetal development, playing a key role in reducing GDM risk and minimizing its associated complications [8]. A balanced macronutrient composition includes carbohydrates, which are vital for providing energy, maintaining stable maternal glucose levels, and supporting fetal brain development; proteins, which also contribute to the growth of fetal tissues, placenta formation, and maternal muscle maintenance [9]; and healthy fats, in particular omega-3 and omega-6 fatty acids, which are essential for fetal brain and eye development [10]. The role of specific macronutrients in GDM risk is complex, with research presenting a mixed picture [11]. While increased energy intake has been significantly associated with an increased risk for GDM, the role of macronutrients and of the specific foods may be more clinically significant [8,11]. For carbohydrates, the focus has shifted from quantity to quality; diets with a low glycemic index (GI) or glycemic load (GL) appear beneficial in managing GDM [12,13,14]. Additionally, higher intake of dietary fiber, particularly from cereals and fruits, is consistently associated with a significantly lower risk of GDM [15,16]. Evidence regarding dietary fat is largely unfavorable, as multiple meta-analyses link higher intakes of total, animal, and even vegetable fat to an increased GDM risk [17,18,19]. The association of protein is the most nuanced and depends heavily on the food source; higher consumption of animal protein, especially red and processed meats, is consistently linked to a greater risk of GDM [20,21]. Conversely, higher intake of vegetable protein from sources like nuts and legumes is often associated with a significantly lower risk [20,22]. However, this evidence is not entirely uniform, with published data finding no significant association for vegetable protein or even contradictory results for animal protein in different populations [22]. These discrepancies suggest the metabolic effect of a single macronutrient is inseparable from the food it comes from.

These inconsistencies highlight the limitations of traditional nutritional epidemiology, which often evaluates nutrients in isolation. However, people consume foods in complex combinations, not single nutrients, which creates statistical challenges due to high correlations between dietary components (collinearity) and ignores the synergistic effects between them [23,24]. This has led to a shift toward dietary pattern analysis, which examines the effects of the overall diet [25]. While this approach is more holistic, it often fails to identify which specific components within a dietary pattern are driving the health outcome [24]. Thus, while we know certain food combinations are beneficial or harmful, we lack a clear understanding of how the specific mixtures of macronutrients within those foods contribute to GDM risk. Novel mixture approaches, i.e., Weighted Quantile Sum (WQS) regression, allow for simultaneous evaluation of multiple dietary components, assigning weights to individual foods that contribute most strongly to an overall nutrient mixture [26]. This provides a more realistic picture of how combinations of foods, rather than single nutrients, shape GDM risk. While a few studies have applied such approaches during pregnancy [27,28], little is known about the role of pre-pregnancy diet, particularly in relation to maternal body mass index (BMI) status.

The aim of this study was to address this gap using WQS regression, a novel statistical method designed for mixture analysis. This approach can assess the joint effect of a complex macronutrient mixture while simultaneously identifying and quantifying the importance of each individual component. By analyzing macronutrients as a mixture, we seek to determine which components are most strongly associated with either an increased or decreased risk of GDM. We hypothesized that higher pre-pregnancy exposure to energy and macronutrient mixtures dominated by processed/energy-dense foods would be associated with increased odds of GDM

## 2. Materials and Methods

### 2.1. Study Design and Population Characteristics

This current study is part of the BORN2020 project, a large, prospective, population-based cohort study which commenced in Thessaloniki in 2020 (ethics decision no: 6.231/29 July 2020) and ran between January 2020 and January 2024. Its aim was to gather and analyze information regarding the physical and nutritional status of pregnant women in Northern Greece. Participants were recruited during their routine first-trimester prenatal care visit, between 11^+0^ and 13^+6^ weeks of gestation, at the 3rd Department of Obstetrics and Gynecology, School of Medicine, Faculty of Health Sciences, Aristotle University of Thessaloniki, Greece. Prior to participation, all women were fully informed about the study and provided written consent.

The inclusion criteria required participants to be women aged 18 years or older, with a singleton ongoing pregnancy and sufficient knowledge of the Greek language. Women were excluded if they had multiple pregnancies, a specific nutritional program due to health conditions (e.g., malabsorption syndrome, inflammatory bowel disease), or if they had been diagnosed with certain pre-existing medical conditions, such as type 1 or type 2 diabetes mellitus, chronic hypertension, renal disease, or autoimmune disorders. Additionally, women with incomplete or missing essential data were also excluded from the study.

### 2.2. Dietary Assessment

Participants completed a structured food-frequency questionnaire (FFQ) tailored to the study population [29], which captured usual intake patterns across pre-pregnancy for up to six months before (e.g., previous weeks or months). Respondents indicated how often they consumed specified items, ranging from “never” to multiple times per day, and typically also estimated portion sizes, either through standard measures or provided visual aids. Dietary data from the FFQ were entered into NutriSurvey (EBISpro, Willstätt, Germany, https://www.nutrisurvey.de/index.html (accessed on 13 October 2025)). For items not available in the national tables, data were complemented using the USDA national nutrient database.

### 2.3. Maternal Variables and Covariates

Maternal characteristics with established relevance to gestational diabetes mellitus were collected and incorporated into the analyses. Maternal age and parity were included as demographic and obstetric factors consistently associated with altered glucose metabolism and pregnancy outcomes. Pre-pregnancy body mass index was treated as a principal variable and an effect modifier, given its strong predictive value for gestational diabetes and its potential to interact with dietary exposures. Smoking status and thyroid disease were considered due to their documented effects on endocrine and metabolic regulation, which may influence gestational glucose tolerance. Total energy intake and physical activity were included as lifestyle covariates to account for variation in caloric balance and insulin sensitivity. The selection of these variables was determined a priori, based on epidemiological evidence and biological plausibility, to reduce residual confounding and strengthen the interpretability of the dietary mixture models.

### 2.4. Diagnosis of GDM

GDM was diagnosed following the guidelines of the Hellenic Society of Obstetricians and Gynecologists, which align with the thresholds derived from the HAPO study [30]. All women underwent a 75 g oral glucose tolerance test (OGTT) after an overnight fast and a three-day carbohydrate-rich diet; GDM was defined by the presence of any one of the following glucose levels: fasting ≥ 92 mg/dL, 1 h ≥ 180 mg/dL, or 2 h ≥ 153 mg/dL.

### 2.5. Statistical Analysis

To investigate the relationship between macronutrient intake from dietary sources and GDM, WQS regression models were applied. The analysis focused on the effects of macronutrient mixtures derived from food sources, aiming to identify whether certain combinations of dietary macronutrients were associated with increased or decreased risk of GDM.

The dietary intake of each macronutrient was estimated from FFQ data. For each food item, the average content of the respective macronutrient per standard portion was computed using nutrient composition data. These values were then multiplied by the reported daily frequency of consumption for each participant to obtain food-specific macronutrient intake variables. This procedure was executed for the pre-pregnancy period, which was for up to six months prior to conception, generating a comprehensive set of macronutrient exposures.

WQS regression was performed using the food-specific intake variables as mixture components for each macronutrient. The outcome variables were binary indicators of GDM. The WQS index was constructed using quantiles of the macronutrient intake distributions, and model estimation involved repeated bootstrapping to obtain stable weights for the contribution of each food source to the mixture. In this context, the term mixture refers to the combined intake of all food items contributing to a given macronutrient or energy source, with each food assigned a weight representing its relative contribution to the overall association with GDM risk. The analysis adjusted for a consistent set of potential confounders: parity (nulliparous vs. multiparous), maternal age, pre-pregnancy BMI, thyroid disorders, and smoking status. Energy intake and physical activity were also included as period-specific covariates.

Models were run separately for each macronutrient. The primary output of interest was the adjusted odds ratio for the WQS index, interpreted as the change in odds of the outcome associated with increasing exposure to the mixture. In addition, normalized weights were derived to estimate the relative contribution of each food source to the overall mixture effect. This allowed the identification of the foods most responsible for the observed association between macronutrient intake and GDM risk.

Detailed food-specific weights for each macronutrient mixture are provided in the Supplementary Material, while the main text presents the most relevant contributors to facilitate interpretation (Supplementary Datasets S1 and S2).

## 3. Results

A total of 797 women were included in the final analysis. Table 1 shows characteristics of the included population stratified by GDM status.

Table 2 presents the associations between macronutrient-specific WQS indices and GDM risk in normal-weight and overweight/obese women. Among normal-BMI women, several macronutrient indices were significantly associated with increased risk of GDM. Higher energy intake was strongly related to GDM (aOR = 81.16, 95% CI: 4.55–1447.46, *p* = 0.003), as were total carbohydrates (aOR = 50.66, 95% CI: 3.59–715.04, *p* = 0.004). Total fat (aOR = 9.35, 95% CI: 1.17–74.54, *p* = 0.035) and total protein (aOR = 11.06, 95% CI: 1.14–107.09, *p* = 0.038) were also significant, indicating that both higher fat and protein mixture exposures contributed to GDM risk. In contrast, dietary fiber (aOR = 3.01, 95% CI: 0.52–17.41, *p* = 0.219), vegetable protein (aOR = 5.92, 95% CI: 0.81–43.29, *p* = 0.080), and animal protein (aOR = 3.98, 95% CI: 0.68–23.39, *p* = 0.100) showed non-significant associations.

Among overweight and obese women, no significant associations were observed across any macronutrient indices. Energy (aOR = 0.39, 95% CI: 0.003–47.56, *p* = 0.70), total carbohydrates (aOR = 1.28, 95% CI: 0.023–70.05, *p* = 0.90), dietary fiber (aOR = 0.83, 95% CI: 0.073–9.48, *p* = 0.88), total fat (aOR = 0.21, 95% CI: 0.004–9.68, *p* = 0.42), and total protein (aOR = 0.98, 95% CI: 0.016–60.39, *p* = 0.99) all showed null associations, as did vegetable protein (aOR = 1.24, 95% CI: 0.056–27.44, *p* = 0.89) and animal protein (aOR = 1.03, 95% CI: 0.13–8.00, *p* = 0.98).

Table 3 shows the overall mixture effects of macronutrient intake in normal-weight women pre-pregnancy. In women of normal weight, the energy mixture was significantly associated with GDM risk (*p* = 0.003, aOR = 81.16, 95% CI 4.55–1447.46). The foods that contributed most to this effect were olive oil (6.2%, *p* < 0.001), sugar and honey in beverages (6.0%, *p* < 0.001), puff pastry (5.5%, *p* < 0.001), low-fat and fermented dairy products (5.0%, *p* < 0.001), and eggs (4.7%, *p* < 0.001). Additional contributors included tea (4.3%, *p* < 0.001), dried fruits (3.3%, *p* < 0.001), full-fat dairy (2.8%, *p* < 0.001), whole-grain bread (2.5%, *p* = 0.0187), olives (1.5%, *p* = 0.0324), and whole-grain pasta (1.4%, *p* = 0.0461).

For carbohydrates, the mixture was strongly associated with GDM (*p* = 0.004, aOR = 50.66, 95% CI 3.59–715.04). The largest contributors were puff pastry (9.1%, *p* < 0.001), potatoes (6.0%, *p* < 0.001), and eggs (5.6%, *p* < 0.001). Additional significant foods included sweets (4.9%, *p* = 0.006), red meat (2.8%, *p* = 0.007), fresh fruits (2.6%, *p* = 0.003), whole-grain bread (2.4%, *p* = 0.041), and boiled salad (2.3%, *p* = 0.042).

The dietary fiber mixture was not significant (*p* = 0.2191, aOR = 3.01, 95% CI 0.52–17.41). Despite this, several individual foods carried notable weight with very low *p*-values, such as processed meat (14.9%, *p* < 0.001), potatoes (14.1%, *p* < 0.001), and puff pastry (11.7%, *p* = 0.0295). Still, these results should be considered exploratory.

The fat mixture was significantly associated with increased GDM risk (*p* = 0.035, aOR = 9.35, 95% CI 1.17–74.54). Key contributors were puff pastry (10.1%, *p* = 0.0017), processed meat (8.9%, *p* < 0.001), low-fat and fermented dairy products (8.2%, *p* < 0.001), potatoes (6.8%, *p* < 0.001), eggs (7%, *p* = 0.0453), olive oil (6.8%, *p* < 0.001), red meat (4.2%, *p* < 0.001), full-fat dairy (3.6%, *p* < 0.001), orzo (3.3%, *p* = 0.0185), full fat whipping cream (1.9%, *p* = 0.0351), and whole grain pasta (1.9%, *p* = 0.0351).

The overall protein mixture was also significant (*p* = 0.038, aOR = 11.06, 95% CI 1.14–107.09). The strongest contributors were low-fat and fermented dairy (9.9%, *p* < 0.001), processed meat (7.9%, *p* < 0.001), puff pastry (7.3%, *p* < 0.001), tea (6.4%, *p* < 0.001), eggs (6.4%, *p* = 0.007), full-fat dairy (3.9%, *p* = 0.001), canned juice (3.1%, *p* = 0.03), orzo (2.5%, *p* = 0.007), olives (2.3%, *p* = 0.04), and cereals (1.9%, *p* = 0.03).

The vegetable protein mixture showed a borderline association with GDM (*p* = 0.079, aOR = 5.92, 95% CI 0.81–43.29). Significant contributors included potatoes (13.7%, *p* < 0.001), processed meat (11.5%, *p* < 0.001), puff pastry (10.2%, *p* < 0.001), and tea (6.8%, *p* < 0.001), alongside smaller contributions from whole-grain bread (6.4%, *p* = 0.003), alcohol (5.6%, *p* = 0.002), and nuts (3.9%, *p* = 0.028).

Finally, the overall mixture of animal protein foods was not significantly associated with GDM risk (*p* = 0.1258, aOR = 3.98, 95% CI 0.68–23.39). Within this group, however, some items had low *p*-values and higher weights, such as puff pastry (weight = 13.8%, *p* < 0.001), low-fat and fermented dairy (13.0%, *p* < 0.001), eggs (10.5%, *p* < 0.001), and whipping cream (low-fat, 5.3%, *p* = 0.0047), though these should be interpreted cautiously since the overall mixture was not significant.

Table 4 displays the full mixture results for overweight and obese women. In overweight and obese women, none of the macronutrient mixtures showed a statistically significant association with GDM risk at the overall level. The energy mixture (*p* = 0.9624, aOR = 1.09, 95% CI 0.04–33.36) was not significantly associated with GDM risk. Nevertheless, several foods contributed disproportionately to the mixture. Puff pastry (8.2%, *p* < 0.001) and cereals (7.2%, *p* < 0.001) carried the highest weights, followed by olives (4.5%, *p* = 0.001), snacks (3.8%, *p* = 0.0040), and fresh fruits (3.7%, *p* = 0.0983). Legumes (3.6%, *p* = 0.0532), white pasta (3.4%, *p* = 0.0711), and lean meat (3.0%, *p* < 0.001) also appeared as relevant contributors. These results indicate that, although no significant association was observed, certain high-weight, low *p*-value items such as puff pastry, cereals, and lean meat played a disproportionate role in shaping the mixture’s contribution.

For the carbohydrate mixture (*p* = 0.4714, aOR = 3.31, 95% CI 0.13–86.35), no significant effect was observed. However, several foods contributed significantly to this mixture. Puff pastry (8.2%, *p* < 0.001), red meat (5.7%, *p* < 0.001), pies (3.2%, *p* = 0.001), olives (6.6%, *p* < 0.001), sugar–honey in beverages (5.5%, *p* = 0.0160), and fresh fruits (4.1%, *p* = 0.0416) emerged as the strongest contributors. These findings indicate that, although total carbohydrate intake as a mixture was not associated with GDM, specific high-weight, high-significance foods were particularly influential.

For the dietary fiber mixture (*p* = 0.9935, aOR = 1.01, 95% CI 0.08–12.39), there was no significant effect. Still, legumes (19.0%, *p* < 0.001), olives (10.9%, *p* = 0.0024), cereals (9.5%, *p* = 0.0016), whole grain bread (8.6%, *p* = 0.0016), puff pastry (8.4%, *p* = 0.0032), and white pasta (5.7%, *p* = 0.0352) significantly contributed. Notably, legumes accounted for almost one-fifth of the weight, suggesting they were a dominant driver of the mixture’s variability, despite the lack of association.

The fat mixture (*p* = 0.2480, aOR = 0.18, 95% CI 0.01–3.27) similarly showed no significant overall effect, yet several individual foods emerged. Puff pastry (8.5%, *p* < 0.001), fish and shellfish (6.8%, *p* < 0.001), olives (6.0%, *p* < 0.001), legumes (6.0%, *p* = 0.0019), red meat (3.5%, *p* = 0.0126), nuts (2.8%, *p* = 0.0106), and snacks (2.8%, *p* = 0.0116) were statistically significant. The high weights of puff pastry, fish, and olives suggest these foods disproportionately influenced the fat mixture’s contribution.

The total protein mixture (*p* = 0.9403, aOR = 0.89, 95% CI 0.04–17.97) also did not reach significance. However, puff pastry (10.1%, *p* < 0.001), legumes (12.4%, *p* < 0.001), and cereals (6.8%, *p* < 0.001) emerged as significant contributors. Low-fat and fermented dairy products (3.7%, *p* = 0.0354) were also statistically significant, while fresh fruits approached significance (2.8%, *p* = 0.098).

The animal protein mixture (*p* = 0.7933, aOR = 0.73, 95% CI 0.07–7.39) showed no overall association with GDM. Still, whole grain pasta (5.9%, *p* = 0.0063), fish and shellfish (5.9%, *p* = 0.0063), processed meat (5.9%, *p* = 0.0063), and eggs (5.9%, *p* = 0.0063) were significant contributors, with lean meat also approaching significance (5.9%, *p* = 0.0410). Despite the uniform weight distribution (each food ~5.9%), these significant *p*-values highlight that some animal protein sources were disproportionately influential.

Finally, the vegetable protein mixture (*p* = 0.8913, aOR = 1.22, 95% CI 0.07–22.12) was not significantly different. Nevertheless, legumes (4%, *p* = 0.0178), olives (4%, *p* = 0.018), and fast food (4%, *p* = 0.0179) were statistically significant contributors, with snacks (4%, *p* = 0.045) and alcohol consumption per day (4%, *p* = 0.0435) also reaching significance.

## 4. Discussion

This study assessed maternal energy and macronutrient intake during the six months preceding pregnancy in relation to the risk of GDM. The findings indicated that (i) higher total energy intake before pregnancy was strongly associated with increased odds of GDM among normal-weight women, with the greatest contributions coming from olive oil, dried fruits, sugar and honey in beverages, puff pastry, low-fat and fermented dairy products, eggs, tea, dried fruits, full-fat dairy, and whole grain bread; (ii) higher total carbohydrate intake was also related to elevated GDM risk in normal-weight women, particularly when carbohydrates were derived from refined or processed sources including puff pastry, red meat dishes, pies, olives, and, to a lesser extent, fresh fruit; (iii) higher total fat intake was significantly associated with increased GDM risk, with processed meats, low-fat dairy products, puff pastry, olive oil, and red meat being the main contributors; (iv) higher total protein intake was similarly linked to greater GDM risk, with low-fat dairy, processed meats, puff pastry, eggs, and tea contributing most strongly; (v) in contrast, dietary fiber, vegetable protein, and animal protein were not significantly associated with GDM risk in normal-weight women; and (vi) in overweight and obese women, none of the macronutrient or energy indices were associated with GDM, suggesting that diet quality in the pre-pregnancy period may be more relevant to GDM development in normal-weight than in overweight women.

Although numerous studies have explored potential etiological links between nutrition and the development of GDM by examining macro- and micronutrient intake, relatively few have investigated the association of specific food groups with the disease. Several studies have suggested that diets characterized by high intakes of total fat and saturated fat, together with lower consumption of carbohydrates, fruits, and vegetables during pregnancy, are associated with an increased risk of developing GDM [31]. Evidence from a study in Spain further indicates that high consumption of potatoes, fast food, and sugar-sweetened beverages prior to pregnancy is independently associated with GDM [32,33]. These food categories did not seem to influence the development of GDM among our population. Some studies have reported no association between total or non-cola sugar-sweetened soft drinks and GDM, suggesting that the observed link with cola beverages may reflect residual confounding or lifestyle-related factors rather than a direct causal effect [34].

Moreover, studies examining food groups and overall dietary patterns have shown that GDM risk is predicted by higher intake of red and processed meats as well as adherence to a Western-type dietary pattern, typically rich in red meat, refined sugars, and fried or snack foods [35]. These findings are similar to ours, as our results showed a tendency to develop GDM with processed meat. Another study suggests that higher consumption of whole grains, fish, full-fat dairy products, and nuts appears to reduce the risk of GDM, whereas red meat and sugar-sweetened beverages are linked to an increased risk, with fruit intake showing a modest protective effect [36]. Carbohydrates from fresh fruit in our study did not seem to have a protective effect. Evidence for other food groups remains inconsistent, although limited data suggest that soybeans and olive oil may also contribute to a lower risk. Overall, high consumption of ultra-processed foods (UPFs) during pregnancy has been positively associated with a range of adverse maternal and child outcomes, including an increased risk of GDM [37].

The effect of carbohydrate consumption on the development of GDM is a main question for many researchers. Meta-analyses have further demonstrated that dietary glycemic index (GI) and glycemic load (GL) are predictive of type 2 diabetes, underscoring the importance of carbohydrate quality rather than quantity. Regarding the contribution of protein to the development of GDM, existing data remain inconsistent. Some studies report no association [38], whereas others suggest a strong influence, showing that higher pre-pregnancy intake of animal protein, particularly red meat, is significantly and positively correlated with the risk of GDM. In contrast, plant protein does not appear to exert the same effect, findings that are consistent with ours [20]. A recent meta-analysis [39] reported that a high-fiber diet is associated with a reduced risk of GDM; however, this association was not observed in our study. Furthermore, previous research has highlighted the potential benefits of dietary fiber supplementation in women with GDM, showing significant improvements in glycolipid metabolism and pregnancy outcomes. In fact, dietary fiber has been proposed as an adjunctive therapy for GDM, with insoluble fiber supplementation being particularly beneficial in cases of poor fasting glucose control [15].

This study has several notable strengths. First, it was conducted within a well-defined longitudinal cohort (BORN 2020), which enabled the detailed collection of dietary, anthropometric, and clinical data over time. The use of a validated food frequency questionnaire strengthened the dietary assessment. This population was routinely and uniformly screened for GDM. Finally, the application of multiple adjusted models that accounted for important confounders such as parity (nulliparous vs. multiparous), maternal age, pre-pregnancy BMI, thyroid disorders, and smoking status provided more reliable estimates of the examined associations. However, certain limitations should be acknowledged. The observational design of the study precludes causal inference. Although adjustments were made for key confounders, residual confounding from unmeasured factors, such as insulin sensitivity or potential dietary reporting bias, cannot be excluded. Moreover, some of the associations, particularly for energy, carbohydrates and protein, were accompanied by wide confidence intervals, indicating limited precision. Therefore, these results should be interpreted with caution, and larger cohorts are needed to validate these results. Furthermore, as the analysis was based on a single regional cohort, the generalizability of the findings may be limited, particularly to populations outside the Mediterranean dietary and cultural context. Finally, dietary intake was assessed only at baseline, and potential changes in diet during pregnancy were not captured, which may have diluted true associations.

## 5. Conclusions

Pre-pregnancy intake of energy and specific macronutrients, particularly when derived from processed and energy-dense food sources, is associated with increased risk of GDM in women of normal weight, but not in overweight or obese women. The role of preconception nutrition in shaping pregnancy outcomes might be a potential window for preventive action. From a clinical perspective, dietary counseling before conception should prioritize moderation of total energy intake and encourage substitution of refined, high-calorie foods with healthier alternatives such as whole grains, fruits, and unprocessed protein sources. Future research should further explore the associations between specific food groups and the development of GDM, using well-designed prospective cohort studies across diverse populations. Pre-pregnancy dietary counseling should be targeted according to maternal BMI. For women of normal weight, reducing consumption of processed and energy-dense foods may represent a key preventive measure against GDM. For women with overweight or obesity, broader strategies addressing baseline metabolic health may be required, as dietary mixtures alone did not significantly alter risk in this group.

## Figures and Tables

**Table 1:** Maternal characteristics of women stratified by GDM status.

Maternal Variables	GDM (Ν = 117)	Non-GDM (Ν = 680)	*p* Value	Comparison
MA (years) (mean, SD)	34.15 (±4.48)	32.1 (±4.89)	*p* < 0.0001	↑ in GDM
MA > 35 years (*n*, %)	51 (43.59%)	186 (27.35%)	*p* < 0.001	↑ in GDM
Weight before pregnancy (median (IQR))	65 (59, 79)	63 (57, 73)	0.008	↑ in GDM
Height (cm) (median (IQR))	165 (161, 170)	165 (162, 170)	0.27	-
BMI before pregnancy (median (IQR))	23.7 (21.7, 28.5)	22.7 (20.8, 26.02)	0.002	↑ in GDM
BMI normal weight pre-pregnancy (*n*, %)	69 (58.97%)	438 (64.41%)	0.31	-
BMI underweight pre-pregnancy (*n*, %)	2 (1.71%)	28 (4.12%)	0.32	-
BMI overweight pre-pregnancy (*n*, %)	21 (17.95%)	139 (20.44%)	0.12	-
BMI obese pre-pregnancy (*n*, %)	25 (21.37%)	75 (11.03%)	0.003	↑ in GDM
Smoking (*n*, %)	21 (17.95%)	60 (8.82%)	0.004	↑ in GDM
Thyroid disease (*n*, %)	13 (11.11%)	93 (13.68%)	0.54	-
Parity (*n*, %)				
0	60 (51.28%)	347 (51.03%)	0.96	-
1	44 (37.61%)	253 (37.21%)	0.98	-
2	12 (10.26%)	69 (10.15%)	0.9	-
3	1 (0.855%)	9 (1.32%)	0.98	-
4	0 (0%)	2 (0.294%)	-	-
Conception with ART (*n*, %)	11 (9.4%)	48 (7.06%)	0.48	-

IQR: interquartile range, represented by the 25th percentile, median, and 75th percentile; n: number of participants; SD: standard deviation; MA: maternal age in years; BMI: body mass index calculated as weight in kilograms divided by height in meters squared (kg/m^2^). Thyroid disease includes conditions such as hypothyroidism, hyperthyroidism, and Hashimoto’s disease. Conception ART refers to conception through assisted reproductive technologies; the “↑” symbol indicates that values in the GDM group are higher than in the non-GDM group. Descriptive statistics include mean (SD), percentiles, or proportions. *p*-values were derived using the *t*-test for normally distributed continuous variables, the Mann–Whitney U test for non-normally distributed variables, and the chi-squared test for categorical variables.

**Table 2:** Association between macronutrient-specific WQS index and risk of GDM in normal-BMI women during pre-pregnancy.

Nutrient	Normal BMI Pre-Pregnancy		Overweight and Obese BMI Pre-Pregnancy	
	aOR (95% CI)	*p*-Value	aOR (95% CI)	*p*-Value
Energy	81.16 (4.55–1447.46)	0.003	0.39 (0.003–47.56)	0.70
Total carbohydrates	50.66 (3.59–715.04)	0.004	1.28 (0.023–70.05)	0.90
Dietary fiber	3.01 (0.52–17.41)	0.219	0.834 (0.073–9.48)	0.88
Total fat	9.35 (1.17–74.54)	0.035	0.208 (0.004–9.68)	0.42
Total protein	11.06 (1.14–107.09)	0.038	0.98 (0.016–60.39)	0.99
Vegetable protein	5.92 (0.81–43.29)	0.080	1.24 (0.056–27.44)	0.89
Animal protein	3.98 (0.68–23.39)	0.1	1.03 (0.131–8.00)	0.98

Values are adjusted odds ratios (aOR) with 95% confidence intervals (CI), estimated from weighted quantile sum (WQS) regression models. Models were adjusted for maternal age, pre-pregnancy BMI, parity, thyroid disorders, smoking status, total energy intake, and physical activity. *p*-values < 0.05 were considered statistically significant. aOR: adjusted odds ratio; CI: confidence interval; WQS: weighted quantile sum; BMI: body mass index.

**Table 3:** Weighted quantile sum regression index weights for each FFQ food item for normal BMI women.

Food Item	Energy (*p* = 0.0027)	Carbohydrates (*p* = 0.0036)	Dietary Fiber (*p* = 0.2191)	Fat (*p* = 0.035)	Protein (*p* = 0.038)	Vegetable Protein (*p* = 0.079)	Animal Protein (*p* = 0.1258)
Puff pastry	<0.0001	<0.0001	0.0295	0.0017	<0.0001	<0.0001	<0.0001
Processed meat	0.076	0.2200	<0.0001	<0.0001	<0.0001	<0.0001	0.0711
Low-fat and fermented dairy	<0.0001	0.0194	-	<0.0001	<0.0001	-	<0.0001
Potatoes	0.2201	<0.0001	<0.0001	<0.0001	0.0662	<0.0001	-
Eggs	<0.0001	<0.0001	-	0.0453	0.0070	-	<0.0001
Tea	<0.0001	-	-	-	<0.0001	<0.0001	-
Whole-grain bread	0.0187	0.0407	0.0277	0.0577	0.0714	0.0033	-
Full-fat and fermented dairy	<0.0001	0.0123	-	0.0001	0.0014	-	0.4992
Whipping cream (full-fat)	0.1999	0.1706	-	0.0351	0.1902	-	0.0358
Whipping cream (low-fat)	0.0461	0.5144	-	0.1536	0.3571	-	0.0047
Red meat	0.1313	0.0072	-	0.0006	0.3381	-	0.0501
Orzo	0.1269	0.1676	0.1350	0.0185	0.0070	-	0.0840
Boiled salad	0.1590	0.0420	0.4717	0.4221	0.7444	0.0869	-
Pies	0.2149	0.1283	0.0754	0.0928	0.0506	0.4693	0.3382
Fresh fruits	0.4929	0.0029	0.3130	0.2504	0.0643	0.1543	-
Dried fruits	<0.0001	0.0119	0.1302	0.2661	0.1355	0.0658	-
Legumes	0.2501	0.5909	0.8423	0.8235	0.5775	0.9222	-
Fast food	0.6038	0.7081	0.8421	0.8465	0.7749	0.8514	0.7943
White bread	0.8341	0.8807	0.8653	0.8158	0.8112	0.8901	-
Veg stew	0.0561	0.1531	0.0525	0.0878	0.2099	0.2157	-
Rice	0.2902	0.2484	0.3424	0.4265	0.3792	0.7612	-
Whole-grain pasta	0.0461	0.1472	0.5793	0.0351	0.1719	-	0.0049
Plant-based dairy	0.2785	0.1414	-	0.3562	0.0729	-	0.0248
Fresh salad	0.3066	0.4581	0.2132	0.3965	0.3700	0.4491	0.3484
Nuts	0.1053	0.0855	0.0260	0.0500	0.3417	0.0283	-
Cereals	0.4946	0.0732	0.0688	0.0487	0.0306	0.4594	-
Olive oil	<0.0001	-	-	<0.0001	-	-	-
Olives	0.0324	0.3468	0.2302	0.1507	0.0402	0.1296	-
Margarine	0.3573	-	-	0.7343	-	-	-
Butter	0.2305	-	-	0.8325	-	-	-
Fish and shellfish	0.8573	-	-	0.7694	0.7479	-	0.4175
Alcohol	0.0233	0.2111	-	-	0.5199	0.0021	-
Sugar and honey in beverages	<0.0001	0.0515	-	-	-	-	-
Sweets	0.0505	0.0062	-	-	-	-	-
Fresh juice	0.7673	0.7261	0.9156	0.8219	0.7376	0.8353	-
Canned juice	0.0681	0.0718	0.1090	-	0.0351	0.1245	-
Lean meat	0.0925	-	-	-	0.1205	-	-
Coffee	0.1608	-	-	-	0.5925	0.2781	-
Coffee decaffeinated	0.7451	-	-	-	0.2590	0.5606	-
Vegetable oils	0.1820	-	-	-	-	-	-
Carbonated drinks with sugar	0.068		-	-	-	-	-
Carbonated drinks without sugar	0.5225	-	-	-	-	-	-
Chamomile and herbs	0.3520	-	-	-	-	-	-
Sweets with sugar alternatives	0.4429	-	-	-	0.4729	0.6765	-
White pasta	0.1199	-	0.4682	-	0.5385	-	0.5174
Snacks	0.1808	-	0.4355	-	0.1309	0.2460	-

*p*-value associated with the estimated weighted quantile sum regression index parameter as given in Table 2. Models were adjusted for maternal age, pre-pregnancy BMI, parity, thyroid disorders, smoking status, total energy intake, and physical activity; BMI: body mass index.

**Table 4:** Weighted quantile sum regression index weights for each FFQ food item among women with pre-pregnancy overweight and obesity.

Food Item	Energy (*p* = 0.9624)	Carbohydrates (*p* = 0.4714)	Dietary Fiber (*p* = 0.9935)	Fat (*p* = 0.2480)	Protein (*p* = 0.9403)	Vegetable Protein (*p* = 0.8913)	Animal Protein (*p* = 0.7933)
Puff pastry	<0.0001	<0.0001	0.0032	<0.0001	<0.0001	0.6711	0.2838
Processed meat	0.2893	0.6391	0.3475	0.1996	0.0682	0.0794	0.0063
Low-fat and fermented dairy	0.8159	0.0522	-	0.4043	0.0354	-	0.3752
Potatoes	0.1265	0.7203	0.3950	0.7855	0.3857	0.2146	-
Eggs	0.2643	0.6030	-	0.3538	0.5051	-	0.0063
Tea	0.0342	-	-	-	0.5327	0.1206	-
Whole-grain bread	0.3214	0.5101	0.0016	0.1140	0.2284	0.0602	-
Full-fat and fermented dairy	0.7205	0.6819	-	0.2239	0.5844	-	0.4016
Whipping cream (full-fat)	0.4278	0.7078	-	0.2459	0.6567	-	0.4054
Whipping cream (low-fat)	0.2187	0.6750	-	0.2940	0.7239	-	0.0510
Red meat	0.1937	<0.0001	-	0.0126	0.2012	-	0.1879
Orzo	0.7420	0.7994	0.7333	0.2037	0.7309	-	0.1190
Boiled salad	0.5802	0.7604	0.8280	0.7119	0.5006	0.0821	
Pies	0.2905	0.0007	0.4712	0.1094	0.3233	0.1562	0.1950
Fresh fruits	0.0983	0.0416	0.4048	0.4119	0.0978	0.0572	-
Dried fruits	0.7882	0.6807	0.6832	0.4549	0.3937	0.4828	-
Legumes	0.0532	0.1263	<0.0001	0.0019	<0.0001	0.0178	-
Fast food	0.2858	0.1515	0.5800	0.1625	0.7319	0.0179	0.2207
White bread	0.6161	0.7429	0.5362	0.2871	0.1705	0.0559	-
Veg stew	0.0724	0.1212	0.4580	0.2560	0.5213	0.2827	-
Rice	0.6321	0.7501	0.7396	0.7578	0.6692	0.1789	-
Whole grain pasta	0.0342	0.6524	0.7939	0.2565	0.4636	-	0.0063
Plant-based dairy	0.7177	0.7331	-	0.4009	0.4334	-	0.2612
Fresh salad	0.8261	0.1001	0.6201	0.1742	0.7467	0.1288	0.1788
Nuts	0.0005	0.0693	0.1721	0.0106	0.3958	0.5228	-
Cereals	<0.0001	0.0573	0.0016	0.1211	<0.0001	0.2222	-
Olive oil	0.1697	-	0.0024	0.2099	0.2176	0.0179	-
Olives	0.0009	0.0006	-	-	-	-	-
Margarine	0.1040	-	-	0.4143	-	-	-
Butter	0.7946	-	-	0.6984	-	-	-
Fish and shellfish	0.0031	-	-	<0.0001	0.2482	-	0.0063
Alcohol	0.0444	0.08635	-	-	0.7667	0.0435	-
Sugar and honey in beverages	0.1031	0.0160	-	-	-	-	-
Sweets	0.7389	0.8868	-	-	-	-	-
Fresh Juice	0.2994	0.7102	-	-	-	-	-
Canned juice	0.7469	0.9165	-	-	-	-	-
Lean meat	0.0001	-	-	-	-	-	-
Coffee	0.3549	0.1853	-	-	-		-
Coffee decaffeinated	0.0342	0.5806	-	-	-	-	-
Vegetable oils	0.0342	-	-	-	-	-	-
Carbonated drinks with sugar	0.0342	0.7586	-	-	-	-	-
Carbonated drinks without sugar	0.0342	0.6324	-	-	-	-	-
Chamomile and herbs	0.3674	0.3810	-	-	-	-	-
Sweets with sugar alternatives	0.5409	0.6888	-	-	-	-	-
White pasta	0.0711	0.1285	-	-	-	-	-
Snacks	0.0040	0.0834	-	-	-	-	-

*p*-value associated with the estimated weighted quantile sum regression index parameter as given in Table 2. Models were adjusted for maternal age, pre-pregnancy BMI, parity, thyroid disorders, smoking status, total energy intake, and physical activity.

## Data Availability

Data are unavailable due to privacy restrictions concerning patient confidentiality.

## Supplementary Materials

The following supporting information can be downloaded at: https://www.mdpi.com/article/10.3390/nu17203256/s1, Supplementary Dataset S1: Detailed food-specific weights for energy and macronutrient mixtures in relation to GDM risk for normal BMI women; Supplementary Dataset S2: Detailed food-specific weights for energy and macronutrient mixtures in relation to GDM risk for overweight and obese women.
